# Evodiamine Inhibits Colorectal Cancer Growth via RTKs Mediated PI3K/AKT/p53 Signaling Pathway

**DOI:** 10.7150/jca.92087

**Published:** 2024-03-04

**Authors:** Qiao Zheng, Shengnan Jing, Lin Hu, Xiangrui Meng

**Affiliations:** 1 Infectious Diseases, Hospital of Chengdu University of Traditional Chinese Medicine, Chengdu 611137, China.; 2 Traditional Chinese Medicine (TCM) Regulating Metabolic Diseases Key Laboratory of Sichuan Province, Hospital of Chengdu University of Traditional Chinese Medicine, Chengdu 611137, China.; 3 Clinical School of Medicine, Chengdu University of Traditional Chinese Medicine, Chengdu 611137, China.

**Keywords:** Evodiamine, Colorectal cancer, RTKs, PI3K/AKT/p53, Natural product

## Abstract

**Objective:** To investigate the inhibitory effect of EVO on colorectal cancer (CRC) growth and further explore the potential mechanism involving the RTKs-mediated PI3K/AKT/p53 signaling pathway.

**Methods:** Firstly, the inhibitory effect of EVO on CRC cells was detected *in vitro* by cell viability assay and colony formation assay. The effects of EVO on spatial migration and invasion capacity of cells were detected by Transwell assay. The effects of EVO on apoptosis and cycle of cells were detected by flow cytometry. Then, the molecular mechanism of EVO against CRC was revealed by qRT-PCR and Western blot. Finally, the excellent anti-tumour activity of EVO was verified by *in vivo* experiments.

**Results:** The results demonstrated that EVO exerts inhibitory effects on CRC cell proliferation, invasion, and colony formation. The cell cycle assay revealed that EVO induces G1/S phase arrest. Through RNA seq, we explored the influence of EVO on the transcriptional profile of colon cancer and observed significant activation of RTKs and the PI3K/AKT pathway, along with its downstream signaling pathways. Furthermore, we observed upregulation of p53 proteins by EVO, which led to the inhibition of Bcl-2 expression and an increase in Bax expression. Consistently, EVO exhibited remarkable suppression of tumor xenograft growth in nude mice.

**Conclusion:** This study confirmed that EVO inhibits the proliferation of CRC cells and promotes cell apoptosis. The possible mechanism of action is inhibiting the expression of the RTK protein family, activating the PI3K/AKT/p53 apoptotic signaling pathway, thereby inhibiting Bcl-2 expression and increasing Bax expression, promoting apoptosis of CRC cells. As a natural product, EVO has very high potential application value.

## Introduction

Colorectal cancer (CRC) is one of the most common digestive malignancies with a poor 5-year prognosis and poor quality of life 1. There were about 1.9 million newly diagnosed cases of CRC worldwide in 2020, according to the International Agency for Research on Cancer (IARC). (the third highest prevalence), while CRC causes an estimated 935,000 deaths (the second highest mortality rate) 2, 3. Current treatments include endoscopic and surgical partial resection, supplemented by radiotherapy, ablation, biologics, and immunotherapy 4. While these treatments improve survival, 60% of patients still have a risk of recurrence and metastasis even after surgical removal 5. To improve outcomes, more studies are underway to identify new treatments and assess their effectiveness.

In recent years, the widespread utilization of traditional Chinese medicine in the management of malignant tumors has highlighted its distinct role in antitumor treatment 6-8. Chinese herbal medicine offers various advantages, including reduced toxicity, enhanced efficacy, decreased tumor recurrence rates, and improved survival outcomes for cancer patients. It holds significant potential to enhance the prognosis of individuals diagnosed with cancer. As research continues to delve into the anti-tumor effects of Chinese herbal medicine, considerable progress has been made in unraveling the pharmacological mechanisms of numerous Chinese herbal medicines and their individual components, which have demonstrated promising anti-tumor effects 9, 10.

Evodiamine (EVO) is an indole alkaloid isolated and purified from the Chinese herb evodia rutaecarpa 11, 12. It has good anti-tumor ability, anti-inflammatory and analgesic effects, protects the myocardium, and lowers blood lipids. At present, many studies have found that EVO can play a role in inhibiting tumor cell proliferation by regulating the activity of various signaling pathways. Liu applied EVO to human gastric cancer SGC-7901 cells and found that apoptosis rate was significantly increased 13. The mechanism that promotes its apoptosis may be by regulating the abnormal expression of Frizzled-1 to inhibit the activation of the Wnt3α/β-catenin signaling pathway and reduce the metastasis of gastric cancer cells 14.

Bioavailability data determines the minimum dosage of a drug for treating diseases. However, the clinical data on the bioavailability of EVO has shown surprising results. In a study by Shyr et al 15, high recovery rates of Evo were observed in plasma and feces samples collected from rats after intravenous injection (1 mg/kg) and oral administration (500 mg/kg). However, other results indicated poor oral bioavailability of EVO demonstrated the inhibition of angiogenesis in liver cancer by suppressing β-catenin-mediated VEGFa regulation in a xenograft mouse model of H22 transplantation 16. This inhibition resulted in the suppression of tumor growth. Similarly, Han et al 17. constructed a xenograft mouse model of breast cancer using 4T1 cells and found that EVO could slow down the proliferation of breast cancer cells by inducing cell cycle arrest at the G2/S phase. Although current research findings have shown that EVO has an anticancer effect on CRC by activating the PI3K/AKT signaling pathway, there is still insufficient detailed research data to clearly demonstrate the molecular mechanism or targets of EVO in CRC treatment.

**Figure 1:**
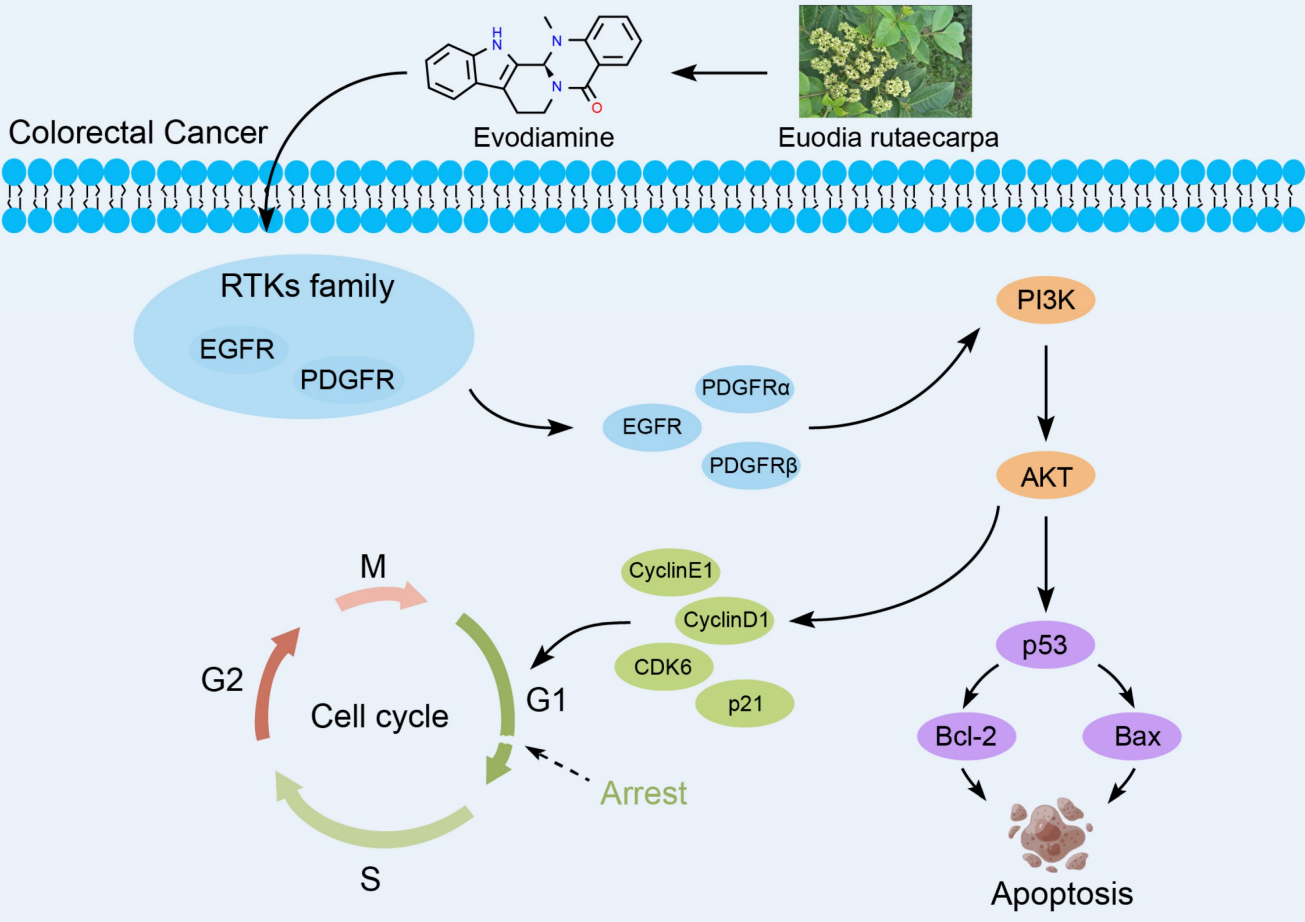
Biological mechanism of evodiamine inhibiting the growth of colorectal cancer.

Receptor tyrosine kinases (RTKs) are enzymes that catalyze phosphorylation of downstream target proteins and play an essential role in a range of cellular processes such as cell growth, differentiation and metabolism. Disorders in RTKs signaling pathways lead to tumorigenesis 17-19. Many studies have shown that targeting EGFR in the anti-RTKs family may be an excellent strategy for treating CRC 20, 21. This article explores the inhibitory effects of EVO on CRC *in vitro* and *in vivo*, and mainly reveals the potential biological mechanisms. First, *in vitro* experiments confirm that EVO has the ability to inhibit proliferation, promote apoptosis, inhibit migration and invasion of CRC cells. After EVO intervention, apoptosis can be induced by regulating Caspase-9 and PARP proteins. Tumor metastasis can be inhibited by inhibiting the EMT process and MMP9 expression. In addition, EVO downregulates the expression levels of RTKs family proteins, while acting on the PI3K/AKT apoptotic pathway, and finally regulates the downstream p53 pathway to exert antitumor activity. For the first time, this study explored the molecular mechanism of EVO against CRC upstream and downstream of the PI3K/AKT pathway. It is hoped that this will provide new ideas for the development of EVO and related agents (Figure 1).

## Results

### Evodiamine inhibits colorectal cancer cells proliferation

**Figure 2:**
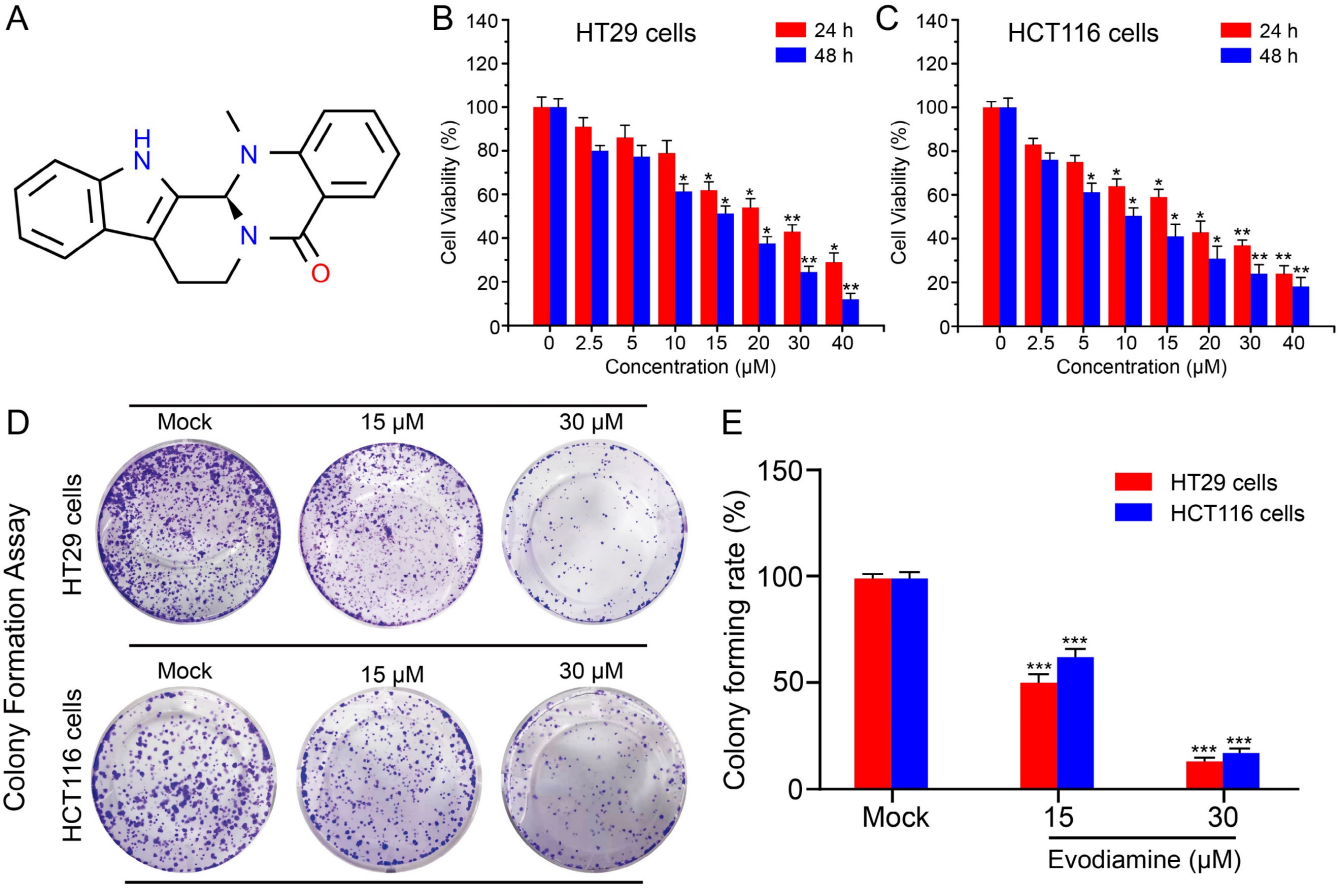
** Evodiamine inhibits the proliferation of colorectal cancer cells.** (A) The chemical structure of Evodiamine. Cell viability of colorectal cancer HT29 cells (B) and HCT116 cells (C) treated with different concentrations of EVO (0, 2.5, 5, 10, 15, 20, 30, 40 μM) for different times (24 h and 48 h). (D) Digital photographs of colony formation experiments of HT29 and HCT116 cells. (E) Quantitative analysis of the number of cells in the colony formation experiments. All experiments were conducted three times independently. *P < 0.05, **P < 0.01, and ***P < 0.001 vs Mock.

EVO, derived from the plant Evodia rutaecarpa, is an indole alkaloid and a natural medicinal compound. The chemical structure of EVO (Figure 2A) consists of a pyridine ring and an epoxy ring, with an aromatic ring positioned between these two rings. On the pyridine ring, there is an amino group and a methyl group, while the epoxy ring bears a hydroxyl group and a methyl group. This unique structure grants EVO with diverse biological activities, including anti-inflammatory, anti-tumor, and antibacterial effects. Hence, we aim to investigate the potential anti-tumor properties of EVO against CRC through both *in vitro* and *in vivo* experiments.

First, we selected the CRC cell lines HT29 and HCT116 cells to study the cytotoxicity of EVO. Using the classical CCK-8 method, we detected the anti-proliferative activity of different concentrations of EVO (0-50 μM) and different time points (24 h and 48 h) on CRC cells. As shown in Figure 2B and C, EVO can significantly inhibit the proliferation of HT29 and HCT116 cells in a concentration-dependent manner compared to the control group. After treatment of the two cells for 24 h, the IC50 was 30 μM (HT29) and 15 μM (HCT116), respectively. After treatment of the two cells for 48 h, the IC50 was 15 μM. Therefore, in subsequent *in vitro* experiments we will treat the cells with 15 and 30 μM. The anti-proliferative effect was then verified by colony formation assay. As shown in Figure 2D and E, EVO treatment significantly reduced the number of colony formations compared to the control group, and the results decreased with increasing EVO concentration. The results confirm that EVO can significantly inhibit the proliferation of CRC cells *in vitro*.

### EVO induces apoptosis of CRC cells

**Figure 3:**
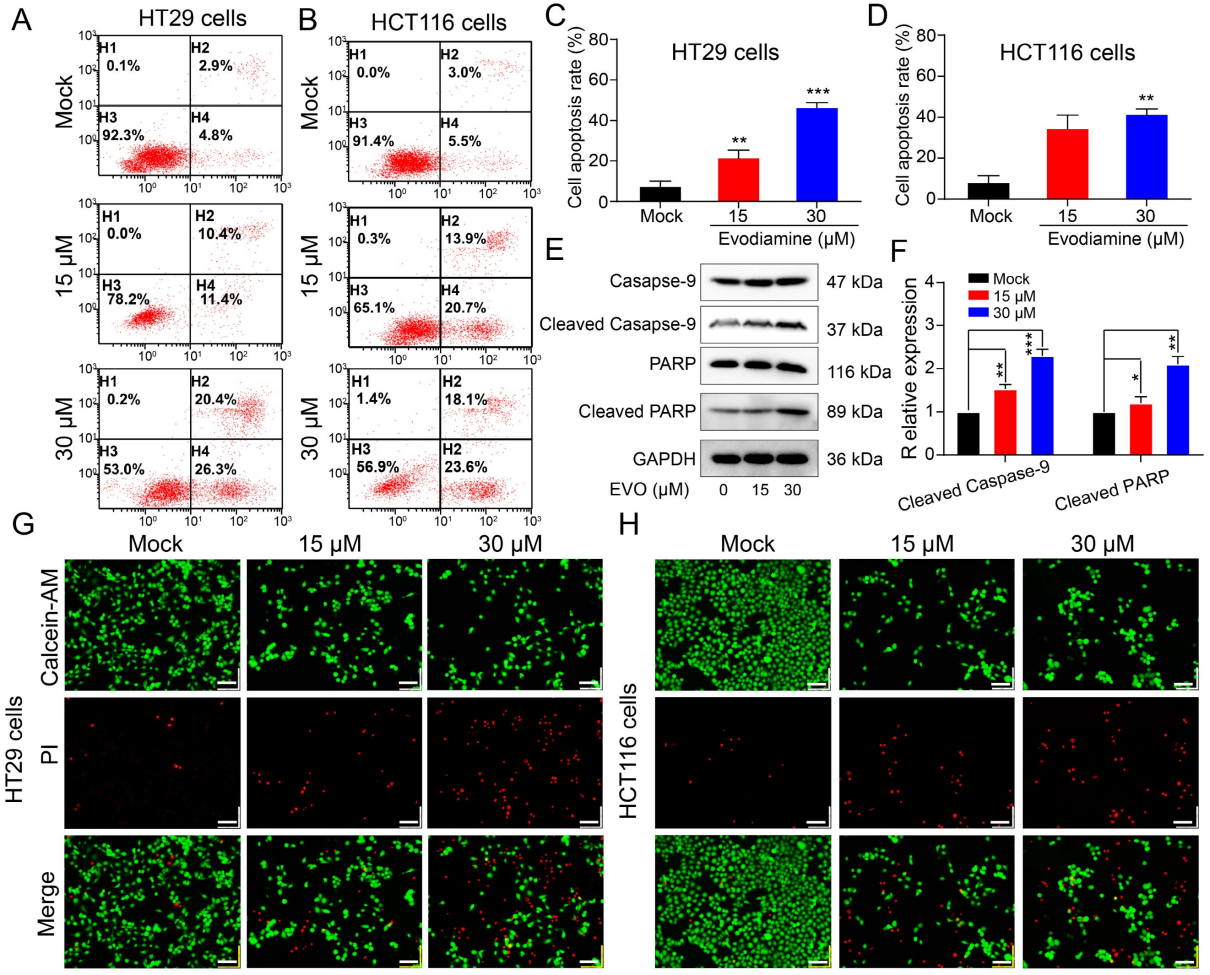
** Evodiamine induces apoptosis of colorectal cancer cells.** Apoptosis of HT29 cells (A) and HCT116 cells (B). Cells were treated with different concentrations of EVO (15 and 30 μM) or an equal amount of DMSO for 48 h and apoptosis was detected using the Annexin V-FITC/PI double staining apoptosis kit. Quantitative analysis of the apoptosis ratio of HT29 cells (C) and HCT116 cells (D). (E) Western blot analysis of apoptosis-related proteins. The expression levels of Caspase-9, Cleaved Caspase-9, PARP and Cleaved PARP were detected after treatment with different concentrations of EVO or an equal amount of DMSO (F). Fluorescence images of HT29 cells (G) and HCT116 cells (H) at 20X magnifications. Live cells (green fluorescence) and dead cells (red fluorescence) were detected using the Calcein-AM/PI assay kit. *P < 0.05, **P < 0.01, and ***P < 0.001 vs Mock.

Apoptosis is a fundamental biological phenomenon that is ubiquitous in the biological world. To further investigate the cytotoxicity of EVO on CRC cells, we used flow cytometry to demonstrate the effects of EVO on CRC cell apoptosis. First, cells were co-incubated with EVO for 48 h and then double stained with Annexin V-FITC/PI. As shown in Figure 3A, B, C and D, HT29 and HCT116 cells were treated with different concentrations (15 and 30 μM) of EVO. The apoptotic rates of the higher concentration (30 μM) EVO group were 46.7% and 41.7%, respectively. Compared to the control group, EVO induced apoptosis in colon cancer cells in a concentration-dependent manner. We then used Western blot (WB) experiments to verify that EVO can promote apoptosis of CRC cells. Caspase (cysteine-requiring aspartate protease) is a family of proteases that play an important role in cell apoptosis. Caspase-9 is an important caspase in tumour cell apoptosis signal transduction. Activation of caspase-9 may be regulated by phosphorylation. WB results (Figure 3E and F) showed that caspase-9 expression levels did not change, but phosphorylated caspase-9 expression increased significantly, indicating that EVO promotes cell apoptosis by affecting the caspase family. PARP is a cleavage substrate of caspase and plays an important role in cell apoptosis. The WB results also confirmed that EVO may affect phosphorylated PARP and these results are statistically significant.

Finally, we observed changes in live and dead cells before and after EVO treatment using confocal laser scanning microscopy (CLSM). Calcein-AM can cause live cells to show green fluorescence, and PI can cause dead cells to show red fluorescence. As shown in Figure 3G and H, 30 μM EVO showed a wide range of red fluorescence, confirming that EVO can significantly promote tumor cell death.

### EVO inhibits the spatial migration and invasive ability of CRC cells

**Figure 4:**
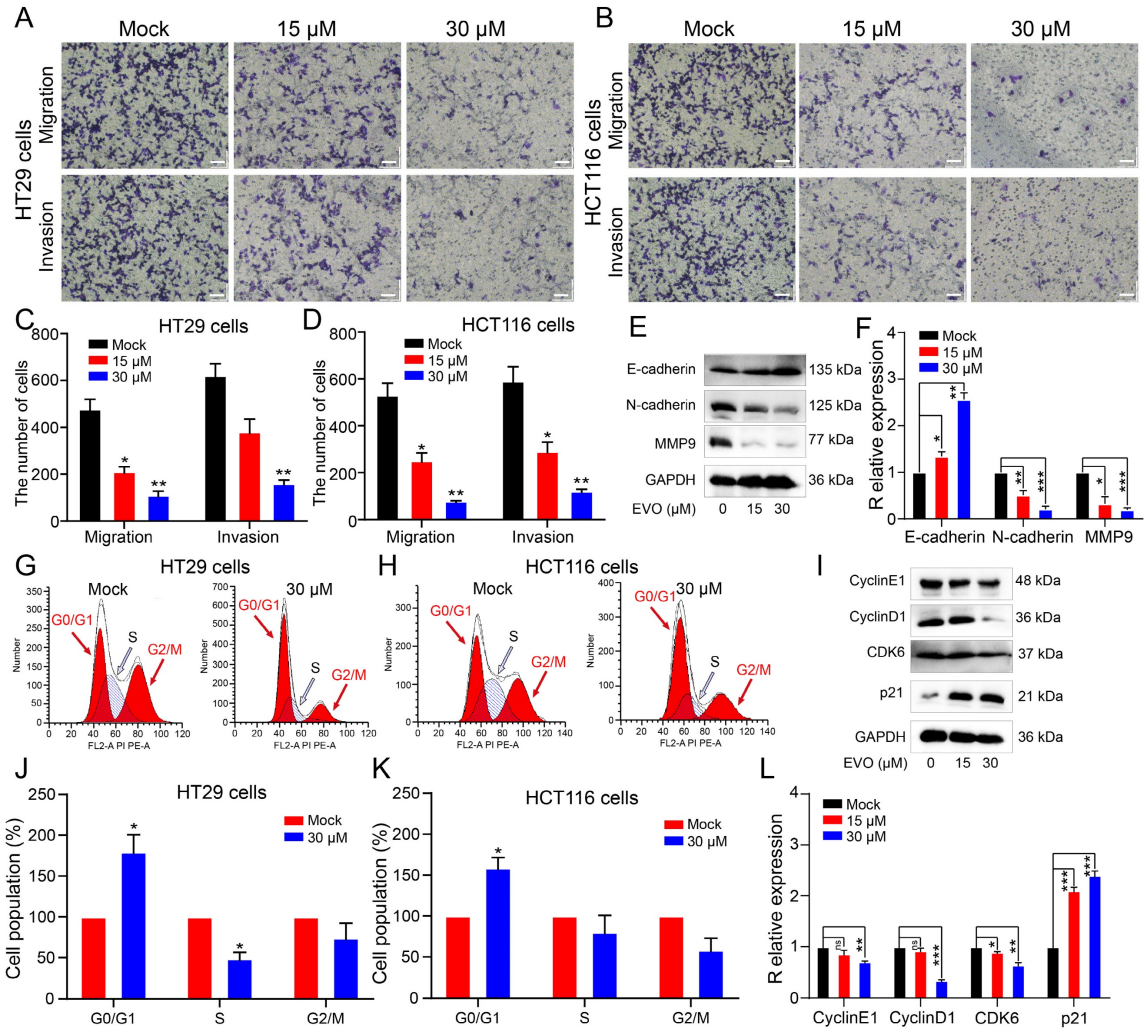
** Evodiamine inhibits the migration and invasion ability of colorectal cells in space and induces G0/G1 phase arrest of colorectal cancer cells.** Migration and invasion ability of HT29 cells (A) and HCT116 cells (B) at 20X magnifications. Quantitative analysis of migration and invasion number of HT29 cells (C) and HCT116 cells (D) after 48 h of treatment with different concentrations of EVO. (E) Western blot analysis of the expression levels of migration and invasion-related proteins. The expression levels of E-cadherin, N-cadherin and MMP9 were detected after 48 h of treatment with different concentrations of EVO or an equal amount of DMSO (F). Flow cytometry detected changes in the cell cycle of HT29 cells (G) and HCT116 cells (H). (I) Western blot analysis of the expression levels of cell cycle-related proteins. Quantitative analysis of the cell cycle of HT29 cells (J) and HCT116 cells (K). (L) Western blot analysis of the expression levels of Cyclin E1, Cyclin D1, CDK6 and P21. *P < 0.05, **P < 0.01, and ***P < 0.001 vs Mock.

Invasion and migration of tumor cells are malignant behaviors of tumor cells. Therefore, we used Transwell experiments to investigate the effect of EVO on the spatial metastatic capacity of CRC cells. As shown in Figure 4A, B, C and D, we treated HT29 cells and HCT116 cells with 15μM and 30μM EVO for 48 h. The results showed that the number of cells stained with crystal violet decreased significantly in a concentration-dependent manner. Epithelial-mesenchymal transition (EMT) is a key step in embryonic development and carcinogenesis 22. The EMT process is most closely associated with the MMP family, which is known to be a major signaling pathway and target for cancer cell migration and invasion 23, 24. Therefore, we used WB experiments to detect the expression of proteins involved in the EMT process and the MMPs family (Figure 4E and F). As expected, after EVO treatment of cells, E-cadherin expression increased, N-cadherin expression decreased, and MMP9 protein expression decreased. The above results confirm once again that EVO has the ability to inhibit spatial metastasis of CRC cells.

To demonstrate the impact of EVO on the lateral migration ability of cells, we conducted a scratch assay to verify the results. The results revealed (Figure S1) that EVO significantly inhibits the migration ability of HT29 and HCT116 cells. Figures S1A and C demonstrate that with increasing concentrations of EVO, the migration ability of HT29 cells is four times weaker compared to the mock group (P<0.05). EVO also exhibits the same effect on HCT116 cells (Figures S1B and D, P<0.01).

### EVO can interfere with the cell cycle of CRC cells

Cancer cells can multiply indefinitely. If the proliferation of cancer cells can be inhibited by interfering with the cell cycle of cancer cells, this will also be a way to inhibit tumor growth^25^, ^26^. Therefore, we used flow cytometry to test whether EVO could interfere with the cell cycle of CRC cells. As shown in Figure 4G, H, J and K, after EVO treatment, the percentage of cells in the G1 phase increased and the percentage of cells in the S phase decreased. We then looked at protein expression levels associated with the G1 phase of the cell cycle to see if EVO could affect the cell cycle. Cyclin E1, cyclin D1, CDK6 and P21 are often referred to as G1-phase regulators. The results showed (Figure 4I and L) that after EVO treatment, Cyclin E1, Cyclin D1 and CDK6 showed a clear downward trend, and the expression level of p21 increased significantly. Conclusion EVO inhibits CRC growth by inducing G1/S cell transition by targeting G1 regulatory factors.

### EVO regulates the biological activity of CRC cells by inhibiting the RTKs/AKT/p53 signaling pathway

To determine the biological mechanism of EVO in inhibiting proliferation and promoting apoptosis in CRC cells, we first performed RNA sequencing (RNA seq) on HT29 cells after EVO treatment to find differentially expressed genes.

**Figure 5:**
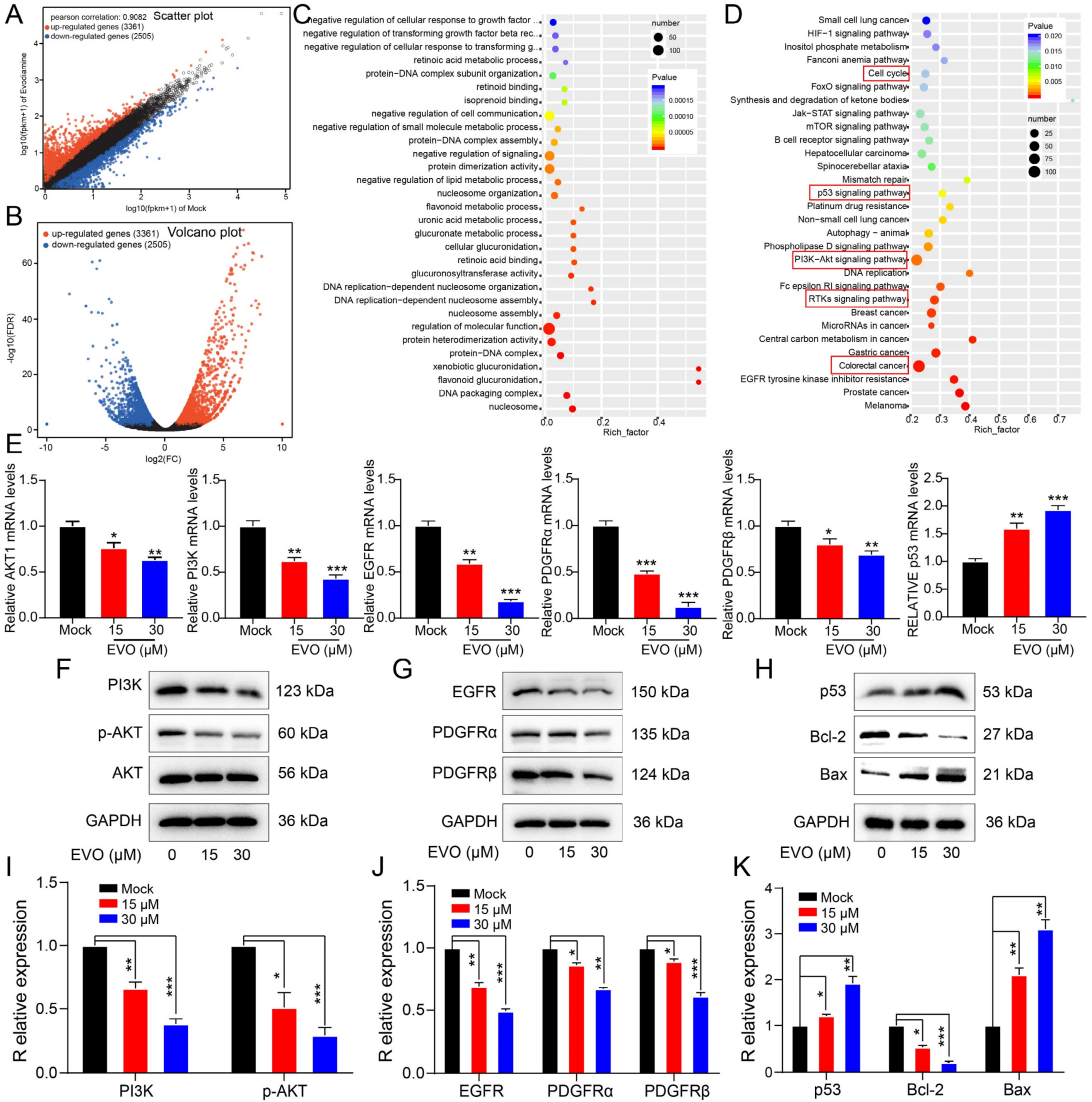
** Evodiamine can inhibit RTKs family proteins, activate the PI3K/AKT/p53 apoptotic pathway, inhibit Bcl-2, and activate Bax.** (A) and (B) Volcano plot analysis of RNA seq sequencing after EVO treatment of HT29 cells. (C) GO enrichment analysis of RNA seq sequencing. (D) KEGG enrichment analysis of RNA seq sequencing. (E) Relative expression levels of AKT1, PI3K, EGFR, PDGFRα, PDGFRβ and p53 mRNA in HT29 cells. Western blot analysis of the expression levels of PI3K/AKT-related proteins (F), RTKs family proteins (G), p53 protein and downstream apoptotic proteins (H). (I-K) Quantification results of PI3K, total AKT, p-AKT, EGFR, PDGFRα, PDGFRβ, p53, Bcl-2 and Bax proteins. *P < 0.05, **P < 0.01, and ***P < 0.001 vs Mock

As shown in Figure 5A, B and Figure S2 there were approximately 3361 upregulated genes and 2505 downregulated genes after EVO treatment. Through Gene Ontology (GO, Figure 5C) and Kyoto Encyclopedia of Genes and Genomes (KEGG, Figure 5D) enrichment analysis, PI3K/AKT, p53 and receptor tyrosine kinases (RTKs) signaling pathways are the most likely biological pathways. Additionally, biological processes indicated involvement of the cell cycle. Predicted diseases encompass CRC. Consequently, we initially validated the expression levels of pertinent differentially expressed genes using quantitative real-time polymerase chain reaction (qRT-PCR), such as AKT1, PI3K, EGFR, PDGFRα, PDGFRβ, and p53. Figure 5E demonstrates a significant downregulation in the expression levels of AKT1, PI3K, EGFR, PDGFRα, and PDGFRβ, while the expression level of p53 exhibited a notable upregulation. Tentatively, PCR results suggest that EVO primarily modulates the expression of receptor tyrosine kinases (RTKs) and the PI3K/AKT pathway, consequently inhibiting tumor growth. Subsequently, Western blot (WB) experiments were employed to further corroborate this hypothesis.

By detecting these indicators at the protein level, the biological mechanism can be more clearly and accurately determined. As shown in Figure 5F and I, we first detected protein expression levels of the PI3K/AKT pathway. The protein expression levels of PI3K and AKT were not different, but the expression levels of phosphorylated PI3K and phosphorylated AKT proteins were significantly downregulated. This suggests that EVO inhibits tumor cell growth through the PI3K/AKT pathway. Next, we identified regulators upstream of the PI3K/AKT pathway. Epidermal growth factor receptor (EGFR), platelet-derived growth factor receptor alpha (PDGFRα) and PDGFRβ have been reported as growth factors in the receptor tyrosine kinase (RTK) family^27^, ^28^. The WB results (Figure 5G and J) showed that the protein expression levels of EGFR, PDGFRα and PDGFRβ were significantly downregulated after EVO treatment. Next, RNA seq results indicated that downstream pathways may be regulated by p53. As predicted, p53 protein expression levels were significantly increased after EVO treatment (Figure 5H and K). Finally, expression levels of p53-regulated apoptotic proteins B-cell lymphoma 2 (Bcl-2) and Bcl-2-associated X protein (Bax) were detected. Bcl-2 expression was significantly decreased and Bax expression was significantly increased. In conclusion, the RTKs /PI3K/AKT/p53 signaling pathway plays a key role in EVO inhibition of CRC growth.

### EVO exerts anti-tumor effects in *in vivo* animal models

*In vitro* experiments have confirmed that EVO can significantly inhibit the activity of CRC cells. To further evaluate the antitumor effect of EVO on animal models *in vivo*, HCT116 cells were subcutaneously xenografted into Bagg albino (BALB)/c nude mice. The EVO group was intraperitoneal injected with 10 mg/kg EVO, and the mock group was injected with an equal amount of saline. During the experiment, the body weight and tumor volume of the mice were observed, and digital photos were taken.

**Figure 6:**
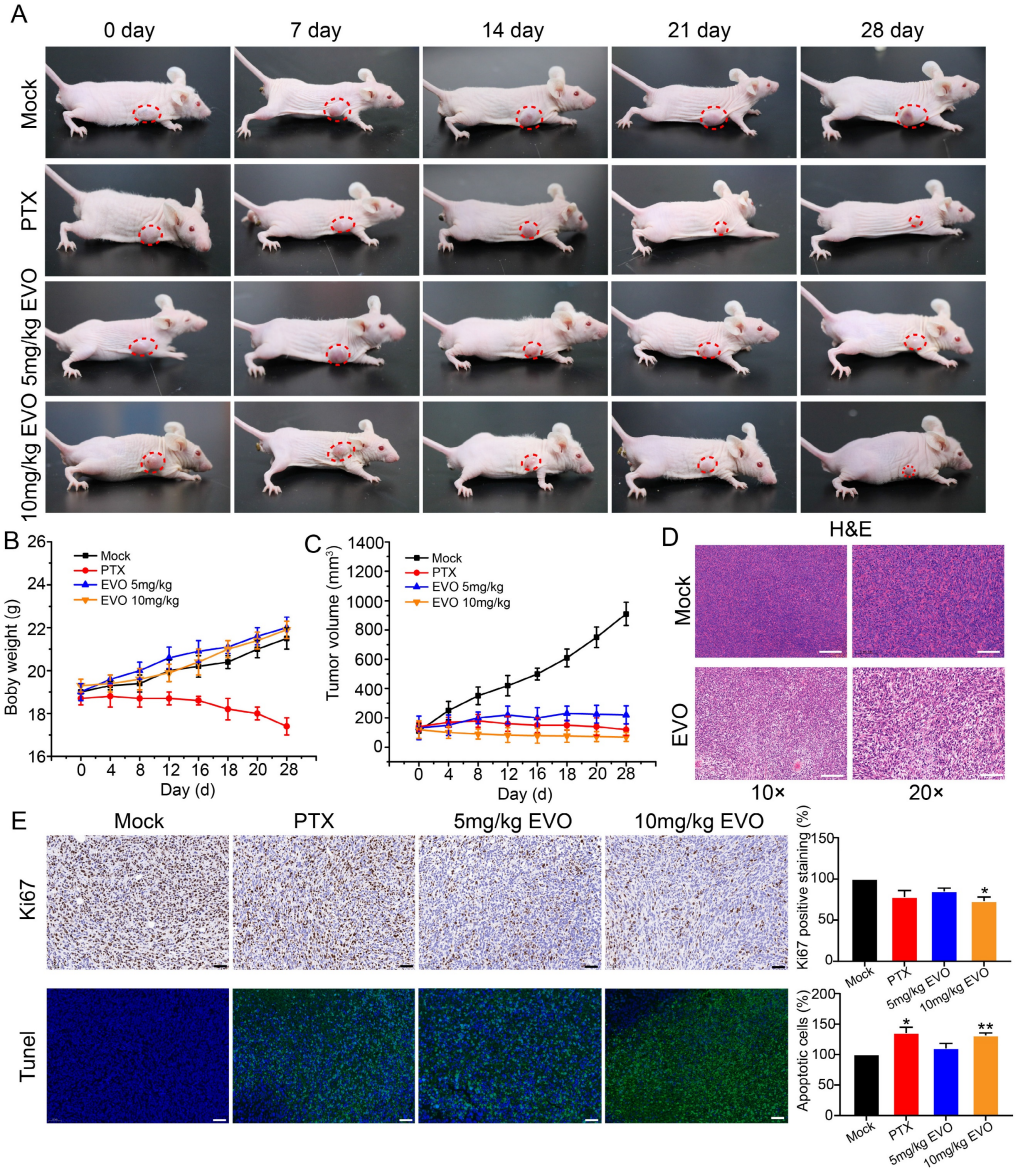
** Evodiamine inhibits tumor growth in a mouse model of colorectal cancer.** (A) Digital photos of changes in tumor mouse models during treatment. (B) Changes in mouse body weight during treatment. (C) Changes in mouse tumor volume during treatment. (D) HE staining images of tumor tissues from each group of mice, enlarged 10X and 20X, respectively. (E) Immunohistochemical images of Ki67 in tumor tissues from each group of mice, enlarged 5X, 10X and 20X, respectively. (F) Tunel staining images of tumor tissues from each group of mice, green indicates apoptotic cells, enlarged 5X, 10X and 20X, respectively.

Firstly, paclitaxel (PTX) was used as a positive drug for comparison. After treatment, the treatment group showed a significant inhibition of tumor growth compared to the control group. The PTX group exhibited a trend of tumor growth inhibition over time. However, treatment with PTX as a medication resulted in changes in appetite and body weight in mice, indicating significant toxic side effects (Figure 6A and B). After treatment, compared with the control group, the tumor size of mice in the EVO group was significantly reduced (Figure 6A). Notably, there was no significant difference in the average body weight of mice after EVO administration (Figure 6B). Tumor volume was significantly reduced in the first few days of EVO administration, and subsequent effects of inhibiting tumor growth were observed (Figure 6C). Subsequently, hematoxylin and eosin (HE, Figure 6D) staining were performed on tumor tissues, which was consistent with the results of *in vitro* experiments. Compared to the control group, the expression level of the cell proliferation marker Ki-67 (Figure 6E) was significantly decreased, and the terminal deoxynucleotidyl transferase dUTP nick-end labeling (Tunel, Figure 6E) analysis showed a significant increase in apoptotic cells within the tissue (green fluorescence). PTX is known to have hepatotoxicity, but the histopathological staining results of various organs in mice after EVO treatment also showed low toxic side effects (Figure S3). In summary, EVO can inhibit the growth of CRC *in vivo*.

## Discussion

CRC is a common malignant tumor of the gastrointestinal tract, and its onset has a certain delay. In recent years, its incidence and mortality have shown an upward trend year after year^29^. With the increasing number of CRC patients, traditional chemotherapeutic drugs are prone to develop primary or secondary drug resistance, and have more adverse reactions 30. Traditional Chinese medicine has become an indispensable part of the treatment of CRC because of its unique curative effect and fewer side effects. Natural compounds isolated from traditional Chinese medicine have the effect of blocking the cell cycle and show great potential as anti-tumor drugs 31, 32.

Research has demonstrated that EVO, the principal constituent derived from the traditional Chinese herbal medicine Evodia rutaecarpa, exhibits significant anti-tumor activity. Initially, we conducted CCK-8 and colony formation assays to validate the concentration-dependent anti-tumor effects of EVO. Notably, cancer-associated mutations that disrupt cell cycle regulation primarily impede the cells' ability to exit the cell cycle. Treatment with EVO for 48 hours was found to interfere with the cell cycle of CRC cells. Specifically, EVO predominantly arrested cells in the G1 phase, while also inhibiting progression through the S phase. To further confirm these experimental findings, we assessed the expression levels of G1 phase regulatory proteins using WB.

The metastasis of malignant tumors is one of the difficult problems to solve. The EMT process is a key step in embryonic development and carcinogenesis. The EMT process can endow cells with many capacities, such as migration and invasion, stem cell characteristics and immune suppression. However, these abilities are beneficial to cancer cells. With these abilities, they can metastasize. As an initiator of tumor metastasis, once the expression level of E-cadherin decreases, the tumor may metastasize 33. After EVO treatment of CRC cells, E-cadherin expression increased significantly, while N-cadherin expression decreased, inhibiting tumor invasion and migration 34, 35.

Cell apoptosis is a genetically controlled, autonomous and orderly death of cells. It is an active process and a process of death that cells actively pursue to better adapt to the living environment. RTKs are the largest class of receptor tyrosine kinases. They are not only receptors for growth factors but also enzymes that can catalyze phosphorylation of downstream target proteins. RTKs are also upstream targets of the PI3K/AKT signaling pathway and can regulate AKT expression, thus affecting cancer cell proliferation and apoptosis 36, 37. Abnormal activation of receptor proteins in the RTKs family (such as EGFR and PDGFR) is associated with various tumors and is also an important target for tumor drug research. According to the experimental results, EVO can significantly inhibit EGFR and PDGFR expression (P<0.001). This suggests the possibility of EVO being developed into clinical drugs. PI3K/AKT is a signaling pathway closely related to proliferation and apoptosis. Its downstream pathways are also very important. Among them, p53 (predicted result of RNAseq) is the tumor suppressor gene most closely related to human tumors discovered to date. It mainly induces cell apoptosis by transcriptionally activating other pro-apoptotic genes 38, 39. WB results also confirmed this. After 48 h of EVO treatment, p53 expression was upregulated, p53 downregulated Bcl-2 expression, upregulated Bax expression, reduced mitochondrial transmembrane potential, released some pro-apoptotic proteins from mitochondria into the cytoplasm, and induced cell apoptosis.

In summary, this study confirmed that EVO inhibits the proliferation, migration and invasion of CRC cells and promotes cell apoptosis. The possible mechanism of action is inhibiting the expression of the RTK protein family, activating the PI3K/AKT/p53 apoptotic signaling pathway, thereby inhibiting Bcl-2 expression and increasing Bax expression, promoting apoptosis of CRC cells. As a natural product, EVO has very high potential application value. This study also enriched and expanded the scientific insights of traditional Chinese medicine in the treatment of malignant tumors.

## Material and Methods

### Cell culture

The Cell Bank of the Chinese Academy of Sciences (Shanghai, China) provided human CRC HT29 and HCT116 cells, which were grown in DMEM conditions (10% FBS and 1% streptomycin-penicillin). The cells were kept alive in a 37 °C incubator that was 5% CO_2_ and saturated with humidity. Passaging of cells every three days.

### Cell viability

The traditional CCK-8 assay was performed to measure cell viability. A standard coated 96-well plate with a total of 10^4^ cells was seeded, cultivated for 24 hours, and then treated with various doses of EVO for another 24 h. Each experimental group was set with six holes. Then, each well received 10 uL of the CCK-8 reagent and 90 uL of the DMEM medium combination, which were then incubated for 1.5 h at 37 °C in the cell incubator. After that, the absorbance at 450 nm was detected with an enzyme marker (BK-EL10C). The cell survival rate was calculated as follow: survival rate (%) = [experimental OD value / control OD value] × 100%.

### Colony formation

2,000 HT29 and HCT116 cells per well were seeded in 12-well plates, which were then incubated for 12 h at various EVO concentrations. After the second day, the culture was continued with a regular medium for 14 days, with a three-day interval between changes in the solution. Crystal violet solution of 0.4 percent was used to stain the colonies for 20 minutes. To see how clonogenic the cells were, extra crystal violet solution was removed and the cells were then rinsed with PBS buffer solution. Counts of the number of colonies formed were calculated under a microscope.

### Annexin V-FITC/PI double staining

HT29 and HCT116 cells were digested with 0.25 percent trypsin-EDTA for 1 minute after 48 h of treatment before being resuspended in the appropriate PBS. The supernatant was removed after centrifuging a cell suspension in a volume of 200 microliters for five minutes at 1000 rpm. After that, cells were given three 5-minute PBS washes. Then, after suspending the cells in 195 μL of Annexin V-FITC binding buffer, 5 μL of Annexin-V-FITC (20 μg/mL) was gently added into the system above for a 15-minute incubation period on ice. A flow detection tube with 400 μL of PBS was filled with the mixture. Propidium iodide (PI, 50 μg/mL), was added into a tube containing ten microliters for a two-minute incubation period on ice in the dark. In the end, the apoptosis was detected within 30 min by using a flow cytometer (FACS Calibur, BD Biosciences, U.S.A).

### Calcein-AM/PI double staining

As a cell death assay, Calcein-AM/PI double labeling (cat. no. C2015M; Beyotime Institute of Biotechnology) was employed to count both living and dead cells. Briefly, 1× assay buffer was added to the HCT116 and HT29 cells (5×10^5^ cells/ml), and each well was stained for 30 minutes at 37 °C with 2 mM calcein-AM and 4.5 mM PI. A fluorescent microscope was used to scan the images and count the proportion of positive cells (magnification, ×100).

### Transwell migration and invasion assay

A pore size of 8 uM Transwell was selected for migration and invasion experiments. In the upper chamber of Transwell, we added 100 uL of cell suspension and 500 uL of medium containing 10% FBS to the lower chamber and incubated in an incubator at 37 °C for 48 h. After fixation with 4% paraformaldehyde for 20 min, 0.1% crystal violet was added to stain the cells for 20 min. Eventually, we used fluorescent microscopy (OLYMPUS 1X71) to observe the number of cells in the chamber. Unlike the migration assay, the invasion experiment needs 50 μL of diluted Matrigel matrix gel to be pre-added to the upper chamber and placed in a 37 °C incubator for 4 h to solidify into a gel before adding the cell suspension.

### Cell cycle analylsis

The cell cycle state after drug-only therapy was assessed by flow cytometry. Cells were specifically collected at the designated time points and fixed in 70% ethanol on ice for 2 h. After that they were cleaned with PBS before being stained with 50 mg/ml propidium iodide (PI, Sigma) for 30 minutes at 4 °C. The BD FACSCanto II software from BD Bio-Sciences was used for the final assessment (San Jose, CA, USA). From G0 to G1, S, and G2 to M, cell stages were denoted.

### Transcriptional sequencing

According the manufacturer's instructions, high-quality RNA sample (OD260/280=1.8~2.2, OD260/230≥2.0, RIN≥6.5, 28S:18S≥1.0, >10μg) is used to construct sequencing library. Sample labeling, microarray hybridization and washing were carried out following the manufacturer's standard protocols.

### Reverse transcriptional quantitative PCR

Following the instructions, we extracted total RNA samples by TRIzol reagent (Life Technologies, Grand Island, NY, USA). When the RNA was isolated, the concentration of purified RNA was measured by a UV spectrophotometer (Thermo Fisher). Reverse transcription to cDNA was performed in the extracted total RNA using a reverse Transcriptase Kit (RiboBio, Guangzhou, China). For the analysis of mRNA, SYBR Green Master Mix (Life Technologies, Grand Island, NY, USA) was applied to an ABI 7500 system (Applied Biosystems Foster City, CA, USA) at the following conditions: 95 °C for 10 minutes, 60 cycles of 95 °C for 15 seconds, and 60 °C for 1 minute. The primer sequences used in this study are listed in Supplementary Material (Table S1).

### Western blot

The EVO-treated HT29 cells were gathered and 1 × 10^6^ of them in 200 μL RIPA buffer were suspended. The cells were lysed and total protein was extracted from the cells. BCA protein assay (Sigma) was applied to measure the protein concentration. By loading the proteins onto 10% SDS-PAGE, the protein samples were separated by electrophoresis, followed by transferring the proteins on the gel to a PVDF membrane. Protein antigens were blocked with 10% skimmed milk prepared with 0.1% TBST at room temperature for 60 min. The membranes were then incubated overnight at 4 °C with the corresponding primary antibodies, which included p53 (cat.no.AF5258), EGFR (cat.no.AF7449), Bax (cat.no.AF1270), Bcl-2 (cat.no.AF6285), PDGFRα (cat.no.114C307), Caspase-9 (cat.no.AF1213), PDGFRβ (cat.no.AF6954), MMP9 (cat.no.AF5234), N-cadherine (cat.no.AF5237), E-cadherine (cat.no.AF6759), PI3K (cat.no.AF6681), AKT (cat.no.AF7920), p-AKT (10020148), CyclinE1 (cat.no.AF2491), CyclinD1 (cat.no.AF0126), CDK6 (cat.no.AF0114), p21 (cat.no.AF5252), Cleaved Caspase-9 (cat.no.AF1264), Cleaved PARP (cat.no.AF1567) at a dilution ratio of 1:2000. Following three washes with TBST, the secondary antibody was used to incubate for 60 min, and finally the protein signal was shown with ECL luminol.

### Nude mice model

BALB/c nude mice with an age of 4 weeks and a body weight of 18-22 g were provided by Viton Lever Laboratory Animal Technology Co., LTD. The mice were fed in SPF-grade animal barriers at a controlled temperature of 24 °C, with relative humidity maintained at 50% and fresh air changes of 10 times per hour. 5×10^5^ HCT116 cells were injected subcutaneously into the right flank via nude mice to establish a subcutaneous graft tumor model. After 7 days of modeling, the nude mice were randomly divided into control and 10 mg/kg EVO groups, with 5 mice in each group. The EVO treatment was administered 5 days per week, with weight and tumor size measured every 4 days until week 4. Whether the diameter of the mouse tumor grows to 12 mm and whether there is ulcer at the tumor growth point is taken as the criterion of humane endpoint. Both the research team and the veterinary staff monitored animals twice daily. Health was monitored by weight (twice weekly), food and water intake, and general assessment of animal activity, panting, and fur condition. Finally, in order to reduce the pain of the experimental mice, we put the mouse into a clean container and slowly inject carbon dioxide. With the increase of carbon dioxide concentration, animals will slowly die painlessly. No animals were sacrificed or died before the end of the study.

### Statistical analysis

All data were analyzed using the SPSS 21.0 statistical analysis software (IBM SPSS, Armonk, NY, USA). The data are expressed as the mean ± standard deviation. P<0.05 was considered to indicated a statistically significant difference. Each group should have at least 3 replicate wells, and each cell and WB experiment should be repeated at least 3 times.

## Supplementary Material

Supplementary figures and table.
