# Supplementary material for: Evodiamine Inhibits Colorectal Cancer Growth via RTKs Mediated PI3K/AKT/p53 Signaling Pathway
Source: J Cancer. 2024 Mar 4;15(8):2361–72. doi: 10.7150/jca.92087 (PMC10937270; doi:10.7150/jca.92087)
Supplement: Supplementary file 1 — Supplementary figures and table. [file jcav15p2361s1.pdf]

Supplementary Material

Evodiamine Inhibits Colorectal Cancer Growth via RTKs Mediated PI3K/AKT/p53 Signaling Pathway

Qiao Zheng[1,#], Shengnan Jing[1,#], Lin Hu[1,#,*], Xiangrui Meng[2,3,*]

[1] Infectious Diseases, Hospital of Chengdu University of Traditional Chinese Medicine, Chengdu 611137, China

[2] Traditional Chinese Medicine (TCM) Regulating Metabolic Diseases Key Laboratory of Sichuan Province, Hospital of Chengdu University of Traditional Chinese Medicine, Chengdu 611137, China

[3] Clinical School of Medicine, Chengdu University of Traditional Chinese Medicine, Chengdu 611137, China

[#] These authors contributed equally to this work and share the first authorship.

* Corresponding author: Lin. Hu (276334022@qq.com), Xiangrui Meng (mengxr@cdutcm.edu.cn)

Table S1 Primer sequences in experiments

Gene name	Forward (5’-3’)	Reverse (5’-3’)
GAPDH	GCACAGTCAAGGCCGAGAAT	GCCTTCTCCATGGTGGTGAA
p53	CTGAGGTTGGCTCTGACTGT	TCAAAGCTGTTCCGTCCCAG
AKT1	TGGACTACCTGCACTCGGAGAA	TGGACTACCTGCACTCGGAGAA
PI3K	TCTTATTCCAGACGCATTTCCAC	TGTTCCTCTTTAGCACCCTTTCG
EGFR	AACACCCTGGTCTGGAAGTACG	TCGTTGGACAGCCTTCAAGACC
PDGFRα	GACTTTCGCCAAAGTGGAGGAG	AGCCACCGTGAGTTCAGAACGC
PDGFRβ	GAGAGGTCTTATGCCAGGACTC	TCAACAATGATCTCCCTGACC

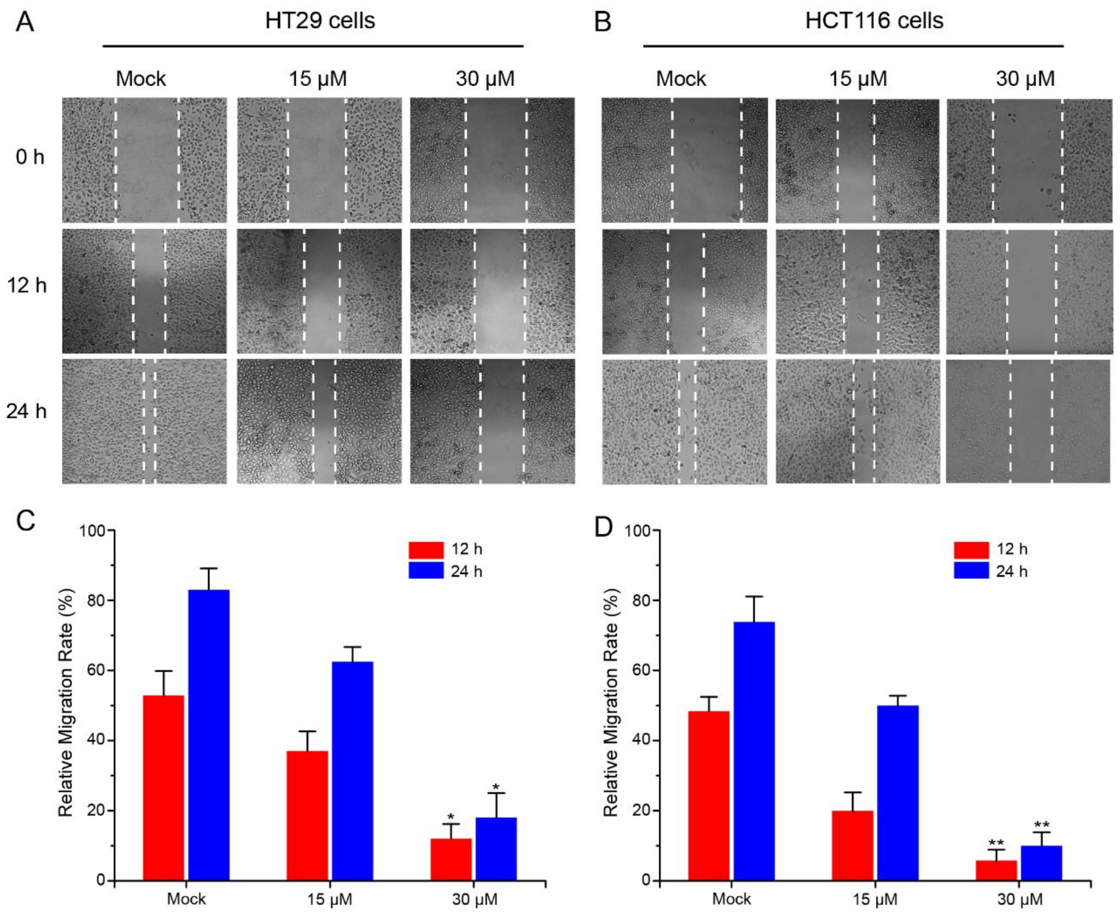

Figure S1. **EVO inhibits the lateral migration of colorectal cells.** Lateral migration ability of HT29 cells (A) and HCT116 cells (B). Quantitative analysis of the lateral migration ratio of HT29 cells (C) and HCT116 cells (D) after treatment with different concentrations of EVO for 12h and 24 h. * $P < 0.05$, ** $P < 0.01$, and ** $P < 0.001$ vs Mock.

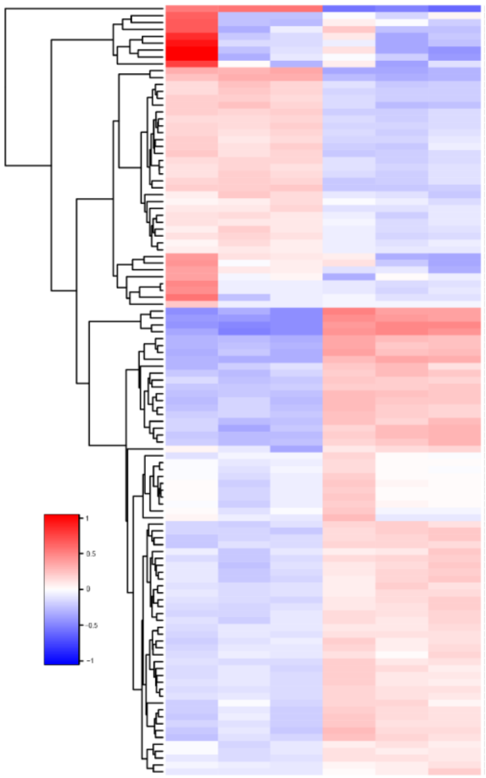

Figure S2. Heat map analysis of differential genes.

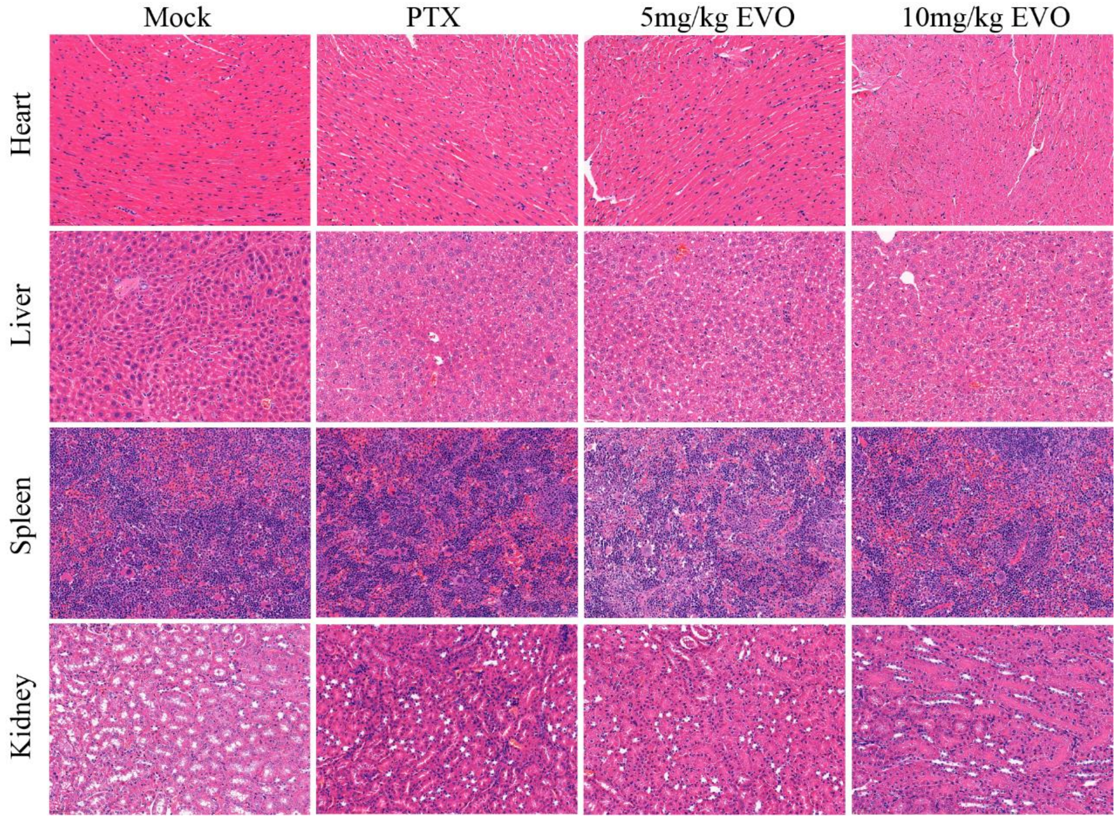

Figure S3. HE *in vivo* tissues under different treatment methods.
